# Split notochord syndrome: a case in point

**DOI:** 10.1590/0100-3984.2015.0251

**Published:** 2018

**Authors:** Camila Soares Moreira de Sousa, Bárbara Bezerra de Castro, Carla Lorena Vasques Mendes de Miranda, Breno Braga Bastos, Marcelo Coelho Avelino

**Affiliations:** 1 Med Imagem - Radiologia, Teresina PI, Brazil.; 2 UDI 24 horas, Teresina, PI, Brazil.; 3 Hospital de Urgência de Teresina Prof. Zenon Rocha, Teresina, PI, Brazil.

**Figure 1:**
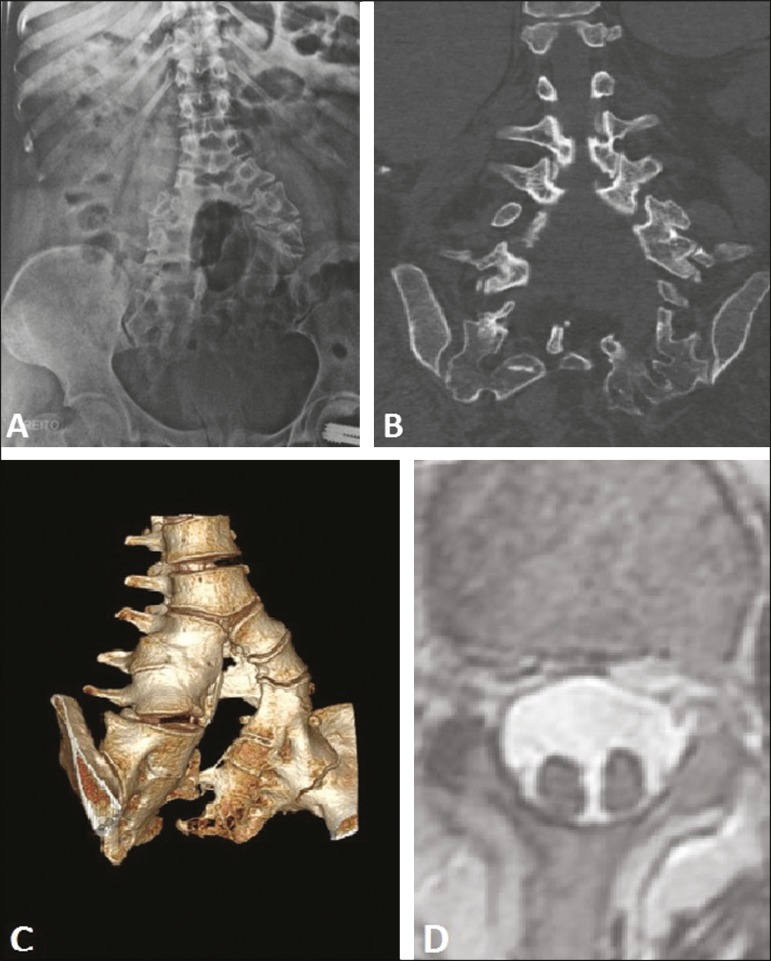
Congenital morphological and structural alteration of lumbosacral spine, characterized by a fusion defect and failure of vertebral body segmentation, with the formation of two axes, starting from the third lumbar vertebra, as seen on conventional radiography (**A**), computed tomography (**B**), and computed tomography with 3D reconstruction (**C**). Axial T2-weighted magnetic resonance imaging scan showing two medullas within a single dural sac (**D**).

Dear Editor,

A 36-year-old female presented with low back pain and was admitted. During the anamnesis, she reported having undergone surgery, in the first days of life, for the repair of lumbosacral myelomeningocele and imperforate anus. She had been a full-term infant, born to an adolescent mother who had received no prenatal care. There were no reports of infection, drug use, or exposure to teratogenic agents during gestation. Computed tomography of the urinary tract showed ectopic kidneys, with a rotational anomaly and bilateral nephrolithiasis. In addition, as an incidental finding, we identified a congenital (morphological and structural) alteration of the lumbosacral spine, characterized by a fusion defect and failure of vertebral body segmentation, with the formation of two axes, starting from the third lumbar vertebra. Other imaging tests for the evaluation of the lumbosacral spine confirmed the findings and demonstrated two medullas within a single dural sac, separated by a thin fibrous septum (Figure 1). These findings, taken together with the clinical history, suggested split notochord syndrome (SNS) as the most likely diagnosis.

SNS results from a rare congenital malformation defined as a cleft in the spine, forming a double spinal column, due to failed fusion of the lateral ossification centers of the vertebral bodies. It occurs most often in the thoracolumbar region; when it affects the lumbosacral region, it can be accompanied by imperforate anus and meningocele. Other anomalies, involving the gastrointestinal tract, central nervous system, and genitourinary tract-manifesting as dorsal enteric fistulas, hydrocephalus, and bladder exstrophy-have been reported^1-3^. Given the scarcity of published cases, it is not possible to accurately establish associations with other abnormalities. An extensive review of the literature showed that approximately 500 cases have been reported and that only a minority of the affected individuals reach adulthood^4-6^.

In most cases of SNS, there are no reports of known exposure to teratogenic agents or a family history of birth defects. Although the embryogenesis of the anomaly remains under discussion, there is evidence that a primary defect in the initial division of the notochord, neurenteric canal, and paraxial mesoderm-with a persistent connection between the endoderm and ectoderm, causing division or deviation of the notochord-is associated with variety of malformations. Recent hypotheses are based on vascular impairment of the inferior neural structure, which would prevent the closure of the neural tube^2,6,7^.

Double spines and spinal cords can be observed in SNS and in caudal duplication syndrome, covering a wide spectrum of malformations, ranging from simple fibrous bands dividing the medulla to complete duplication of the caudal structures. A diagnosis of caudal duplication syndrome should be considered only when there is also duplication of vascular structures or organs, the genitourinary tract, the gastrointestinal tract, and the distal neural tube^8^.

Among individuals with SNS, the reported survival and prognosis are poor. Including the case reported here, there have been only five reports in which the patient survived. However, with the advances in surgical techniques and neonatal intensive care, there is a trend toward better outcomes^2^. The aim of this case report was to discuss the diagnosis of SNS, a rare condition, associated with congenital anomalies and high mortality. Therefore, we propose that, when spinal defects are detected, a detailed investigation of the associated findings be carried out, thus avoiding diagnostic errors and delays.
